# Selecting Appropriate Clinical Trial Endpoints for Geroscience Trials: A Path Towards Consensus

**DOI:** 10.21203/rs.3.rs-5920485/v1

**Published:** 2025-02-12

**Authors:** Stephen Kritchevsky, Ezequiel Zamora, Shalender Bhasin, Alan Cohen, Allison Chandler, Kevin Covinsky, Heidi Crane, Steven Cummings, Kristine Erlandson, Mark Espeland, Sara Espinoza, Luigi Ferrucci, Alexander Fleming, Daniel Forman, Michael LaCroix-Fralish, Mitzi Gonzales, Jamie Justice, Douglas Kiel, George Kuchel, Joan Mannick, Joseph Menetski, Michael Miller, Nicolas Musi, Anne Newman, John Newman, Sarah Simpson Owens, Ambarish Pandey, Kandice Reilly, Mina Sedrak, John Wagner, Heather Whitson, Jeff Williamson

**Affiliations:** Wake Forest School of Medicine; Wake Forest University School of Medicine; Harvard Medical School; Columbia University; Wake Forest University School of Medicine; UCSF; University of Washington; California Pacific Medical Center; University of Colorado-Anschutz Medical Campus; Wake Forest School of Medicine; Cedars Sinai Medical Center; National Institute on Aging; Kitalys Institute; University of Pittsburgh; Marist College; Cedars Sinai Medical Center; XPRIZE Foundation; Hebrew SeniorLife; University of Connecticut Health Center; Tornado Therapeutics; Foundation for the National Institutes of Health; Wake Forest University School of Medicine; University of California; University of Pittsburgh; Buck Institute for Research on Aging; Wake Forest University School of Medicine; Univeristy of Texas Southwestern Medical Center; Wake Forest University School of Medicine; UCLA; Koneksa Health; Duke University Medical Center; Wake Forest School of Medicine

## Abstract

Using a modified Delphi process, we engaged 28 experts in clinical trials, geriatrics, and research translation to determine if there were consensus around what clinical endpoints should be used for trials evaluating the efficacy of interventions to prevent or treat multiple age-related conditions. Four focus groups developed themes. Statements related to those themes were circulated back to participants in a survey. There was consensus (more than 66% agreed or disagreed) that outcome measures should include multiple health dimensions including-age-related disease, function and patient reported outcomes that reflect participants goals; and be tailored to population characteristics. Experts felt that blood-based biomarkers would be unlikely to be accepted as primary endpoints of efficacy trials. Plausible components mentioned as part of a composite endpoint included mortality, mobility function and the onset of multiple age-related diseases. Our findings provide guidance on acceptable approaches to endpoint selection guiding the design of future geroscience trials.

## Introduction

With the global aging of human populations, the burden of chronic health conditions and disability is expected to increase^1^. Since age is the predominant risk factor for most chronic health conditions, one strategy to address this growing societal burden is to modulate the aging process directly^2^. Studies in model organisms suggest that it is possible to modify the aging process and extend lifespan by pharmacologic and nonpharmacologic interventions; these studies have inspired the field of Geroscience which is guided by the hypothesis that targeting the undelying mechanisms of aging can simultaneously slow the onset and progression of multiple age-related health conditions^3^.

The geroscience hypothesis has yet to be tested in rigorously conducted randomized trials^4^. Confirmation of this hypothesis would require the demonstration of reduced mortality from age-related causes and/ or reduced incidence or progression of age-related health conditions. Such trials require large sample sizes and long treatment durations^5^. For example, the Targeting Aging with Metformin (TAME) Trial was designed to determine whether metformin would reduce the onset of several age-related diseases (acute coronary syndromes, decompensated heart failure, most cancers, mild cognitive impairment / dementia, stroke, and death); it was estimated that such a trial would require 3,000 participants treated and followed for 5-years to have adequate power to detect a 20% reduction in the disease composite. The TAME trial’s endpoint was selected based on data available from prior metformin trials and pharmacoepidemiologic observations (https://www.afar.org/tame-trial)^6^. Metformin is an unusual case because it had already been in wide-spread use and extensively studied; a situation that may not apply to promising treatments in the future.

There has been intense interest in building consensus and setting priorities for identifying and validating aging-related biomarkers to guide the development and evaluation of geroscience-inspired therapies^7–9^. An important long-term goal of the field is to identify and validate biomarkers as surrogate endpoints in clinical trials. A surrogate endpoint, as defined by an FDA-NIH working group, is “... a substitute for a direct measure of how a patient feels, functions or survives. Biomarkers may include molecular, histologic, radiographic, or physiologic characteristics. A surrogate endpoint does not measure the clinical benefit of primary interest in and of itself, but rather is expected to predict that clinical benefit or harm based on epidemiologic, therapeutic, pathophysiologic, or other scientific evidence” (https://www.ncbi.nlm.nih.gov/books/NBK326791/). If the FDA accepts that a surrogate endpoint has been validated, drug registration trials can use the surrogate measure to establish treatment efficacy. Absent a surrogate, drug registration trials must be based on the ascertainment of relevant clinical outcomes. The availability of a validated surrogate can mean the difference between large, long expensive trials and smaller, shorter trials. For example, trials evaluating the effects of a new treatment on bone mineral density can be substantially smaller than trials evaluating the effect on the rate of osteoporotic fractures for which bone mineral density is a surrogate^10^. Validation of BMD as a surrogate for skeletal fracture risk is in its final review at FDA (https://www.asbmr.org/about/news-release-detail/fda-issues-timeline-determination-on-fnih-asbmr-sa)^11^. Validation of a biomarker starts with evidence that the biomarker is directly related to the same biological pathway that contributes to the clinical outcome. The FDA expects multiple trials to show the biomarker’s correlation with an FDA recognized clinical outcome, because it must be shown that the biomarker response reliably predicts the clinical benefit and is related to the magnitude of the clinical benefit. itself a daunting task^12^. There are no accepted surrogates of the aging process, and emergence of a surrogate is unlikely until clinical benefit of an intervention has been demonstrated in a randomized controlled trial.

This leads to an important unresolved question in Geroscience: what are suitable primary endpoints for trials that evaluate the efficacy of interventions targeting aging biology? Though dependent on context of use to some degree, the answer is fundamentally important in guiding the design, duration and sample size of such efficacy trials. In the absence of randomized trial data, soliciting the views of scientific, clinical and regulatory stakeholders on plausible trial endpoints would be valuable; better yet, consensus of these stakeholders if it could be reached, would be catalytic in harmonizing outcome data collection and the design of initial smaller trials. A consensus could also enable subsequent data pooling and meta-analyses and help guide the development of better translational animal models and the analyses of observational data from epidemiologic studies for generation of supportive evidence.

To date, there has not been a consensus process focusing on the selection of outcomes with the goal of informing the design of future geroscience-inspired trials to evaluate the efficacy of an array of interventions targeting aging-related pathways; such trials are designed to investigate interventions targeting the biological mechanisms of aging, aiming to delay onset of age-related diseases and enhance healthspan. To begin the consensus building process we engaged experts in aging and geriatrics, clinical trial design, and translational Geroscience to participate in a modified Delphi process. The goal of the process was to identify areas of consensus and disagreements surrounding the specification of clinical outcomes in geroscience clinical trials. Discussions related to the aging process, the desirable attributes of trial endpoints, and the relative merits of several potential endpoints including some that have already been proposed such as incidence and progression of one or more age-related diseases^13^.

## Methods and Measures

### Design

Potential panel members were identified using purposive sampling based on their level of involvement in clinical trial design, clinical management of age-related diseases, and regulatory expertise. Of the 31 invited experts, 28 took part in the focus groups. The study was approved by the Wake Forest institutional review board (IRB).

Virtual focus groups were convened between June 2023 and August 2023. Qualitative and Patient Reported Outcomes (Q-PRO) methods were used to capture expert insights on the aging process and appropriate endpoints for Geroscience clinical trials. These recorded sessions were facilitated by two researchers (moderator and note taker) trained in Q-PRO methodologies. A moderator guide comprising of open-ended and probing questions (Supplement A) was developed to facilitate discussions during the sessions. We used focused groups instead of one-on-one interviews as we recognized the value of promoting exchange and dialogue among experts, providing a platform for a facilitator to clarify areas of alignment (and misalignment) within the group. Following each session, Q-PRO staff debriefed, and audio recordings were transcribed, aggregated, and summarized. The coding process involved assigning thematic codes to statements made during the focus group sessions using a codebook developed for the study. Two Q-PRO personnel independently coded transcripts and discrepancies were discussed to resolve.

Using the themes arising in the focus groups, we developed a REDCap electronic survey to quantify the level of agreement or disagreement related to the themes. This survey had three sections: the aging process, general views about Geroscience trial outcomes, and reactions to specific measures that might serve as a primary outcome or part of an outcome composite (see supplement B). The survey contained statements each of which was rated by panel members using a 5-item Likert scale ranging from 1 (strongly disagree) to 5 (strongly agree). It also allowed for free text comments. Prior to distribution to the expert panel members, the questionnaire was pretested to ensure clarity. Once disseminated to focus group invitees and completed, the survey responses were tabulated to identify areas of consensus and disagreement among the attendees. The Survey was administered from November 13, 2023 to February 9, 2024.

### Data Analysis and Debrief

Focus group transcripts were compared to the audio recordings and edited for accuracy. Two Q-PRO researchers reviewed the transcripts and developed a codebook to capture concepts found in the textual data. The codes focused on areas of interest included in the moderator’s guide but also covered emergent concepts discussed by experts.

Data were managed with ATLAS.ti software (https://atlasti.com). Two Q-PRO researchers independently coded two transcripts at a time and met to compare after each pair of transcripts was coded. The codebook was adjusted, as needed, based on discussions of code meanings and applications. Discrepancies in coding were discussed and resolved iteratively. Segments of text were reviewed by code or groups of codes and summarized. Summaries were synthesized into themes using the principles of reflexive thematic analysis.

The frequencies and means of the survey responses were calculated. The average Likert-scale score and standard deviation was calculated. We defined consensus to have been reached when at least 66% of respondents either agreed or disagreed with a statement regardless of intensity.

All focus group attendees were invited to one of two internet-based debrief sessions. Prior to the session, attendees were sent a qualitative report and the tabulated survey results. At the session, the primary findings were presented and discussed, allowing for participants to provide feedback and impressions on the final report. Reactions helped to inform the discussion section of the manuscript.

## Results

### Participant Characteristics

Table 1 shows the characteristics of the focus group participants. Most had a clinical background, with 77% being clinically trained and 58% actively involved in patient care. Specialties represented included General Internal Medicine, Geriatrics, Cardiology, Endocrinology, Clinical Pharmacology, Neuropsychology, Infectious Disease and Oncology. Most participants (90%) had expertise in the biology of aging, design of clinical trials, and/or the regulatory process of drug approval. Approximately two-thirds were men.

### Topic 1: Defining Aging and The Aging Process

The experts opined that a standardized definition of aging would facilitate the selection of outcomes for clinical trials: “Until we can come to consensus...about what we mean by aging, I don’t think we can come to a consensus on how we would measure that.” (Focus group 2, participant 6)

**Figure 1:**
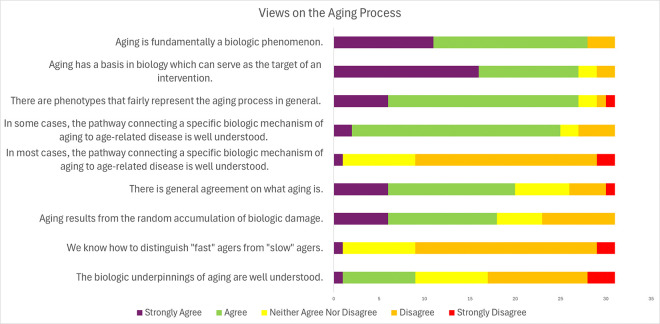
Expert views on the aging process results of a modified Delphi process.

There was strong consensus on several statements regarding the aging process (Table 2; Fig. 1). First, aging is fundamentally a biologic phenomenon (90% strongly agreed or agreed). Second, the biology of aging can serve as the target of an intervention (87% strongly agreed or agreed). Third, there are phenotypes that fairly represent the aging process (87% strongly agreed or agreed). Fourth, in some age-related conditions, the pathway connecting a specific biologic mechanism of aging to age-related disease is well understood (80% strongly agreed or agreed).

However, the survey did not find a consensus on the statement “There is general agreement on what aging is”. Many participants did not think that there was a widely accepted definition for aging. Some experts characterized aging as a multidimensional phenomenon, “at its bare minimum, aging is not one thing. It should be reflected...across systems” (Focus group 2, participant 7) encompassing a spectrum of changes across different systems:

“You could look at any tissue from any organ system and see patterns suggestive of greater biological aging, whether that’s more senescent cells...poor mitochondrial energy, metabolism. In other people’s minds those are the intermediaries. When they talk about aging what they’re really talking about ...is the disease accumulation and worse functioning.” (Focus group 2, participant 1)

There also was not consensus that one could distinguish “fast agers” from “slow agers” when screening for trials targeting the aging process.

### Characteristics of Geroscience Prevention Trial Outcomes

**Figure 3:**
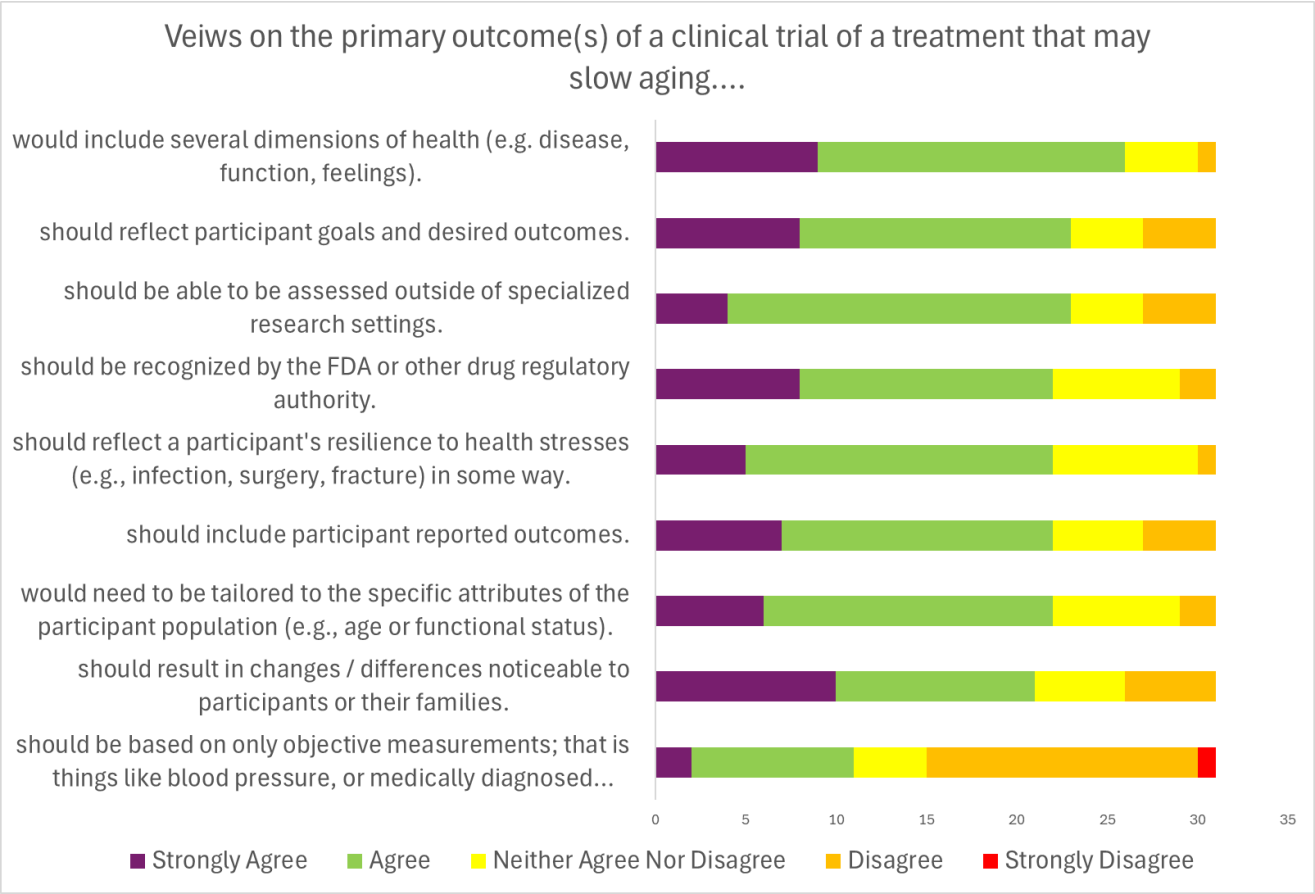
Expert views on components of primary outcomes of a clinical trial of a treatment that may slow aging results of modified Delphi process.

Participants were asked to consider the specific attributes of potential primary outcomes for geroscience prevention trials (Table 4; Fig. 3). There was consensus (84%) that aging measures should include several dimensions of health (e.g. disease, function, patient-reported), reflect participant goals, and be noticeable to participants and their families.

**Figure 4:**
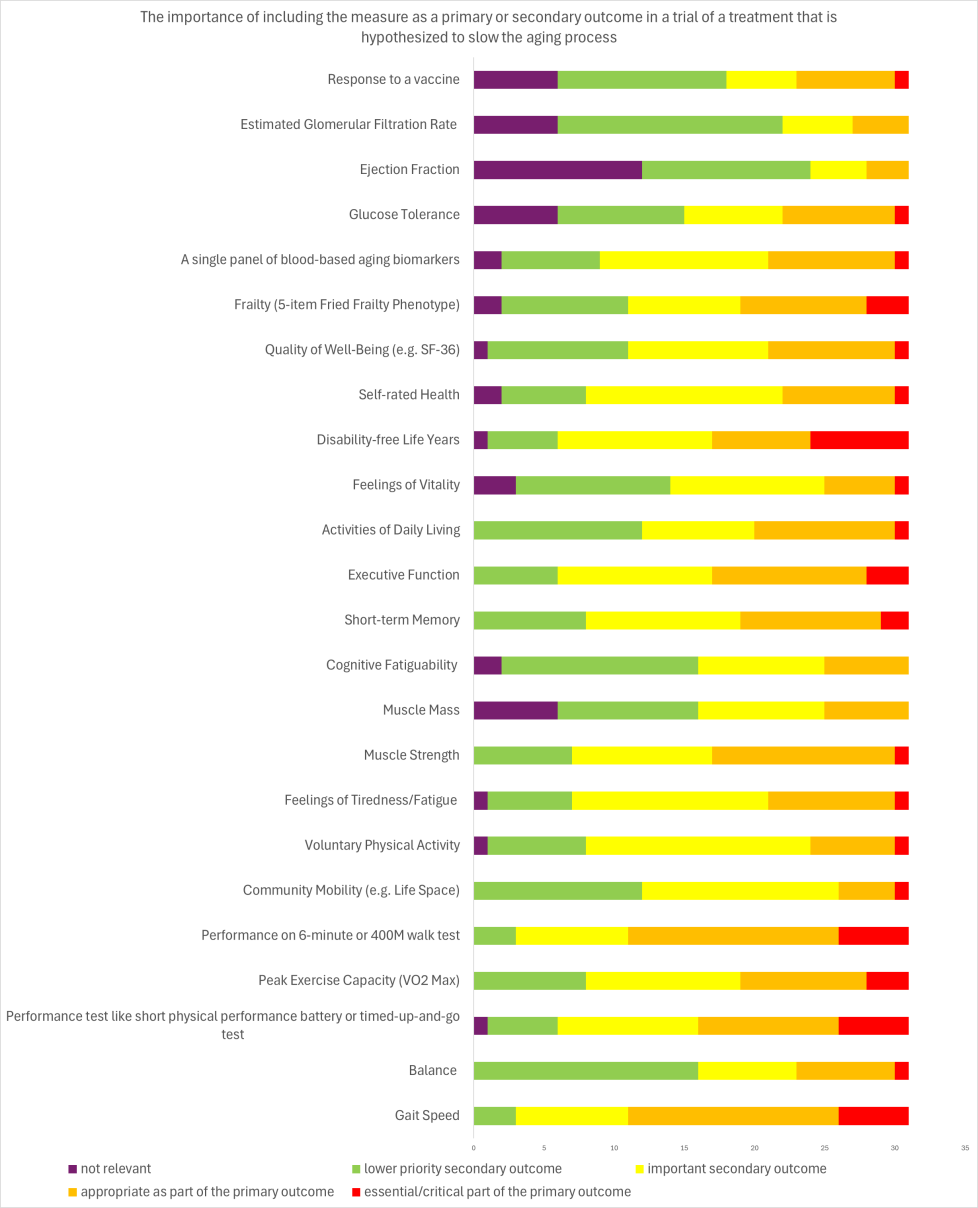
Expert views on endpoint elements or measures of a clinical trial of a treatment that may slow aging results of modified Delphi process.

Furthermore, there was consensus (70%) that aging measures should reflect a participant’s resilience to health stressors, though 25.8% of participants were neutral on this point. Other points of consensus were that the outcome should be assessable outside of specialized research settings, should be recognized by the FDA or other regulatory authorities, and that outcomes would need to be tailored based on the attributes of the participants enrolled. There was strong consensus that outcomes used in different trials should be harmonized to the extent possible (87% strongly agreed or agreed; Fig. 4). Experts discussed various types of outcome measures that might be considered such as patient-reported outcomes (PROs), blood-based biomarkers, measures of physical (e.g., peak VO_2_, and Short Physical Performance Battery) and cognitive function, that could be utilized as endpoints in a study of aging. The experts generally agreed on several concepts: 1) An outcome that combines many potential age-related health conditions reflects the aging process broadly (61% strongly agreed or agreed). 2) An outcome that combines many potential age-related health conditions accounts for the heterogeneity of aging across individuals (55% strongly agreed or agreed). 3) If a treatment slowed the onset of many aging-related diseases that share few risk factors other than age, it would be fair to conclude that the treatment slowed aging (58% strongly agreed or agreed).

PROs were seen as seen as important components of the outcome by many participants.

“Thinking about what is patient important, how to frame the endpoint so that it can be-it’s impact on how the person feels and functions in the home environment. Those PROs are really, really important. As we are thinking about designing these trials, we should also think about designing these PROs that they can validate in early phase two studies.” (Focus group 2, participant 3)

In the survey, there was consensus that outcomes ‘should include participant reported outcomes’ (Table 4). However, there was not consensus for the statement ‘PROs are appropriate as primary outcomes for a trial to test if a treatment slows aging’ (Table 3), There was consensus that we do not have PROs that are widely accepted for use in geroscience trials.

Several experts felt physical function would be a compelling measure given its alignment with aging progression.

“I think the obvious parameters are the classic syndromes of aging...which are best represented by function. So measures of physical and cognitive function...those seem to be the two big ticket items that you would need to show ...that it was slowing the aging process down.” (Focus group 1, participant 6)

Most experts agreed on the importance of including biomarkers in the study of aging. Some experts viewed the utility of biomarkers as an “initial proof of concept” tool. With respect to measures in biologic samples, some experts emphasized “you would [also] want some biosamples, and you would want some performance data.” (Focus group 1, participant 6). There were differing opinions about biomarkers, with a tendency to view them as suitable for secondary outcomes or preliminary measures in early phase trials. A vast majority of experts expressed skepticism about blood-based biomarkers being a sufficient primary endpoint for regulatory agencies, and there was strong consensus (94%) that no single biomarker could definitively demonstrate whether an intervention slowed aging.

### Geroscience Trial Outcome Prioritization

During focus group discussions, experts provided opinions on prioritizing outcomes in Geroscience trials. Functional outcomes emerged as a key topic, “function is very tempting as a clinical outcome... because it is such a powerful predictor” of mortality and other health outcomes (Focus group 3, participant 3). Moreover, PROs, measures of function, and quality of life were discussed as having priority over objective data from biomarkers:

“Anything that directly measures patient reported outcome in context of aging, including frailty burden and physical function status and quality of life, would have a higher...hierarchical status in my mind for an outcome in a study than a biomarker or surrogate measure of aging.” (Focus group 1, participant 1)

However, other experts felt the phase of the trial played a key role in determining the most appropriate endpoints, suggesting early phase trials focus on testing intervention for safety and preliminary efficacy while later phases focus may shift towards functional assessments.

Experts were also asked about frailty without the term being defined by the facilitator. Some regarded frailty to be “another dimension of health [that] is important to measure” (Focus group 4, participant 1), but terms of its prioritization less than half of respondents (39%) felt frailty to be either an essential/critical part or appropriate as part of a primary outcome. One expert offered it would be a “very valuable secondary outcome” (Focus Group 1, participant 6).

Mortality was not felt by most experts to be a high priority, particularly when comparing to function, “mortality reduction is less important than maintaining the other functions that we’ve described...If you want to prioritize the endpoints, I think reduction in mortality may not be number one” (Focus group 2, participant 4) Thirty-eight percent (38%) of experts rated mortality secondary to age-related diseases as either essential/critical or as appropriate as part of the primary outcome.

Discussions also included prioritization of endpoints by regulators (referring to either FDA or “payers”) suggesting entities like the FDA would align their guidance based on “the outcomes that mattered most to people” (Focus group 2, participant 1), like functional status and quality of life.

“The patient reported piece of these measures, which the FDA recognizes as an important outcome, I think is a really important part from the perspective of a participant.” (Focus group 3, participant 6)

Experts also discussed what endpoints would be most valuable to patients. These discussions included the importance of PROs in capturing a patient’s perspective on their health and quality of life. Despite the prominence of PROs in focus group discussions, over half of the experts (55%) indicated neutrality or disagreement on the appropriateness of PROs as primary outcomes for a trial. In addition, most experts (71%) perceive a lack of consensus on which PRO’s would be suitable for inclusion in geroscience-inspired trials. In other discussions, several experts also raised concerns about the potential bias associated with PRO’s. There was strong consensus (87%) among experts that outcomes used in different trials should be harmonized, echoing previous discussions that emphasized the need for standardization of definitions and measures. However, there was no consensus on whether an outcome developed for a trial for one intervention would be appropriate for different interventions.

### Endpoint Selection

During discussions on endpoint selection, experts noted the absence of a universal endpoint and emphasized the alignment of endpoints with the specific research question or type of intervention and its mechanism of action. Critical, and aligned with FDA - can use a qualified Clinical Outcome Assessment (COA) to support a clinical trial endpoint, but appropriateness - and likelihood of subsequent approval - will necessarily depend on the context of use (https://www.fda.gov/about-fda/clinical-outcome-assessment-coa-frequently-asked-questions).

I’d say [an endpoint] will always be tailored, right? There’s not one endpoint that’s relevant. It depends on the intervention... and the population you’re looking at. If it’s maintaining some kind of good health, then that’s one thing. If it’s treating functional decline and trying to improve it, it’s a different kind of thing.” (Focus group 1, Participant 7)

Multiple experts highlighted the complexity and multifaceted nature of the aging process, involving a wide range of biological, physical, cognitive, and social factors. This suggests a dynamic and heterogeneous process that varies among individuals and in which a single endpoint is unlikely to capture all aspects of aging. Most experts also acknowledged that the biological processes underlying aging are interconnected. This gives some credence to the expectation that a selected endpoint used in a trial of a specific targeted intervention could reflect alterations in more than one biological process.

“The endpoint will vary...it depends on what...[you] are proposing you’re changing. If it is something with a functional correlation, then the functional indices we just mentioned will be relevant. If it’s modifying sarcopenia, then I think it’s gonna be an imaging modality. I do think there may be a constellation of biomarkers that are part of everything that we’re talking about, but I think the primary outcome will vary with the question.” (Focus group 4, participant 1)

The survey revealed a strong consensus that a primary endpoint should incorporate several dimensions of health (disease, function, feelings). The majority (72%) also agreed that endpoints should include PROs, reflect resilience to health stresses, changes that participants perceive as being important, and be tailored to the attributes of the study population. There was no consensus on whether primary outcomes should be based solely on objective measures.

### Operationalizing a Geroscience Endpoint

Experts discussed specific proposed measures for studying age-slowing effects, including survival, inflammation, disability/disability-free survival, and function. Functional measures such as the short physical performance battery (SPPB) and peak VO2 were noted to be valuable tools for assessing physical function, fitness and survival prediction.

“Based on the strong observational study’s connection between gait speed and survival and muscle strength and survival, and the SPPB has both those elements...and if you could change those... it does have...attractive features.” (Focus group 1, participant 5)

Experts also discussed the predictive value of inflammatory markers as indicators of a system under stress;

“Inflammation predict[s] a number of things, ...I think that the inflammatory response to chronic inflammation seems to be an expression of a system that is falling apart.” (Focus group 3, participant 7)

Disability and disability-free survival were also considered to be “very impactful endpoints.” One expert proposed a comprehensive definition of disability that included “cognitive or mobility problems” therein “making it a straightforward primary endpoint.” (Focus group 2, participant 3) There was a discussion of the trade-off between outcomes that occur early that would be desirable for prevention RCT but which may be less clear cut as a reflection of aging versus more robust outcomes such as physical and cognitive impairment which may occur later in the aging pathway and may be substantially more difficult to modify after they become clinically manifest.

**Figure 5:**
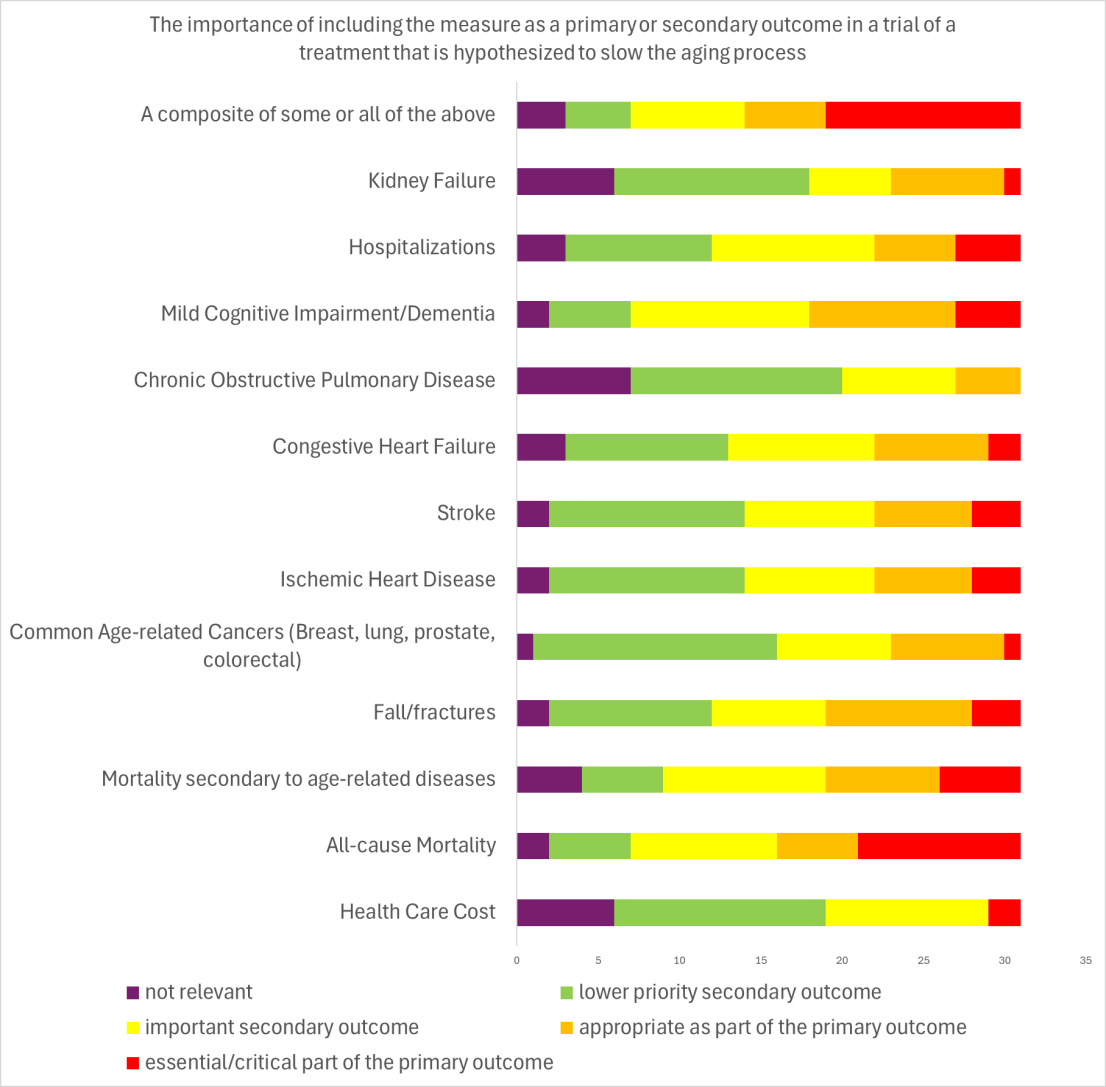
Expert views on the endpoint elements for health-related conditions of a clinical trial of a treatment that may slow aging results of modified Delphi process.

Figures 4 and 5 and supplement Tables 1 and 2 and show survey responses of experts considering which specific measures might be considered as a primary endpoint or part of a primary endpoint, a secondary endpoint, or not relevant, and what the anticipated response to an effective preventive intervention might be. There was no consensus for any specific measure as a standalone or component of a primary outcome. However, most (65%) agreed that a mobility measure like gait speed or 6-minute walk test a suitable primary outcome component, with nearly one-third anticipating improvement of such a measure. While 45% of experts considered muscle strength a plausible component of a primary endpoint, fewer than 20% of experts regarded muscle mass this way. There was no consensus around any specific disease as a key component of a primary outcome. However, 48% endorsed all-cause mortality and 55% agreed that a composite of some diseases and mortality could be a primary outcome. There was consensus among experts (94%) that there was not a single or panel of blood-based aging biomarkers as appropriate primary outcomes though 60% viewed them as appropriate secondary endpoints.

Experts also tended to view fatigue (65%) and cognitive fatiguability (74%) as appropriate secondary outcomes. There was less consensus on cognitive measures such as short-term memory or executive function as opinions varied on whether they should be prioritized as primary or secondary outcomes. However, over half agreed there would be some degree of slowing of cognitive decline if a treatment successfully slowed aging. The majority of respondents (65%) agreed PROs such as self-rated health and quality of well-being were appropriate secondary outcomes.

## Discussion

A prerequisite for testing the geroscience hypothesis is to operationalize the concept of ‘multiple age-related health conditions.’ To date, there has not been a systematic discussion of how to achieve this objective. Here, we engaged leaders involved in clinical aging research to determine if there were a consensus on the most appropriate primary and secondary endpoints for geroscience trials, and, if consensus could not be achieved, to understand the sources of disagreement to help shape future discussions. We focused on the primary endpoint because this measure is pivotal for trial design and informs the eligibility criteria, the sample size, and the study duration. For a Geroscience trial, a meaningful change in the primary endpoint would provide evidence that the aging process was affected in the hypothesized way. Consensus is valuable as it guides the design of pilot studies, observational studies needed to model the endpoint and helps to focus biomarker discovery and validation efforts. While some panel members discussed the role of biomarkers in assessing the efficacy of aging-modulating interventions, these did not play a central role in discussions. We purposely focused on clinical outcomes with the intention of validating biomarkers against these outcomes in future research.

Our focus group discussions and surveys of 28 and 31 experts, respectively, showed consensus that measures should include several dimensions of health (e.g. disease, function, patient-reported), reflect participant goals, and be noticeable to participants and their families. Among the measures mentioned as appropriate as part of a composite endpoint were all-cause mortality, mobility function, and a composite of age-related diseases. Functional measures were discussed as manifestations of the classic syndromes of aging. More than 40% of experts surveyed thought function to be essential or appropriate as a primary outcome without agreement on a single most appropriate measure. Age-related health conditions were also discussed, recognizing that trials of drugs targeting aging-related pathways will be conducted in individuals who, irrespective of intervention and primary outcome, are most likely have co-existing disease and geriatric syndromes of aging. The proposed TAME trial is an example of a Geroscience trial that proposed the use of the incidence of multiple age-related diseases as a primary endpoint based on the effects of metformin on aging biology^6^. The TAME endpoint is a special case of the deficits accumulation view of aging first articulated by Rockwood and Mitinski^14^ which quantifies the percentage of potential health deficits a person has from a predefined list. Deficit indices have not been standardized and can include elements that are not age-related or that may be extensively overlapping. However, the use of this strategy to develop standardized indices tailored to participant characteristics and intervention strategies is consistent with expert sentinment^15^. Nearly half of the experts felt that all-cause mortality was an important primary outcome. Many experts viewed mild cognitive impairment/dementia as an important endpoint in group discussion, but only 42% considered it an essential or appropriate primary outcome.

The lack of strong consensus on the selection of the primary endpoint is attributable to several factors. 1. There is an absence of a universally accepted definition of aging. 2. Endpoint selection is context dependent and largely depends on the phase of the study, the nature of the intervention, and the population being enrolled. Therefore, it is difficult to make universal statements that can apply to all trials in all phases of drug or therapeutic development. One expert noted that early-phase trials might prioritize endpoints testing safety and preliminary efficacy while later phase trials would focus on evaluating functional or clinical disease outcomes. 3. While PROs were considered important, there was a lack of agreement on which PRO would be most appropriate as an outcome measure. 4. Aging is a dynamic and heterogenous process that varies among individuals, making it unlikely that a single endpoint could adequately capture all aspects of its complexity.

Although a consensus on an appropriate primary endpoint was not reached, experts provided guidance regarding important features to consider when constructing a potential endpoint. A multidimensional approach was recommended to capture a broader and more comprehensive picture of how an intervention impacts the complexity of aging. Under this premise, the utilization of composite endpoints was favorably viewed. The utilization of composite endpoints is recognized by the FDA as appropriate in clinical trials where certain disorders have important multiple clinical outcomes which are expected to be impacted by the intervention (https://www.ncbi.nlm.nih.gov/books/NBK326791/). Major Adverse Cardiovascular Event (MACE) is an example of a widely used composite endpoint used in cardiovascular trials (3-point MACE outcome is a composite of nonfatal acute myocardial infarction, nonfatal stroke and cardiovascular mortality)^16^. Composite endpoints offer benefit of reducing sample size and trial duration while maintaining statistical power and avoiding multiplicity created by using multiple endpoints^17^. Using this approach, many expressed the value of incorporating both subjective and objective data to achieve a comprehensive assessment. Additionally, composite measures can allow for hierarchal analyses of the components of the composite endpoint that prioritize patient perspectives by giving more weight to measures like physical function and quality of life. This is consistent with the general agreement that outcome measures would need to be relevant and meaningful to participants, aligning with their health goals and improving quality of life. Analysis can involve comparing proportions between study groups or using time-to-event methods and individual examination of component events should be performed and included in study reports (https://www.fda.gov/media/162416/download)^18^. However, the components of composite must be carefully considered as the treatment’s impact on the composite event rate can be viewed as representing the overall clinical effect when all individual events in the composite endpoint hold similar clinical significance (https://www.fda.gov/media/162416/download). Composite outcomes, if poorly composed, can complicate study execution and data interpretation since it is possible for elements of composites to show conflicting signals in response to a given intervention ^15, 19^.

The role of the FDA was discussed. The FDA’s acceptance of an endpoint was seen as a high priority. However, the FDA’s acceptance is predicated on scientific consensus with supporting empirical findings. COAs play an important role in this process since a qualified COA can be used to support a clinical trial endpoint. COA qualification defined by the FDA as “a regulatory conclusion that FDA finds the COA to be a well-defined and reliable assessment of patient’s symptoms, overall mental state, or how they function” (https://www.fda.gov/about-fda/clinical-outcome-assessment-coa-frequently-asked-questions). An example is the Kansas City Cardiomyopathy questionnaire which is used to evaluate heart failure treatments^20^. However, a COA is only qualified within its specific context of use, thus its appropriateness and likelihood of subsequent approval will depend necessarily on these specific conditions. FDA currently does not recognize aging *per se* as a ‘context of use’ making it difficult to see how to apply this criterion in geroscience research.

Other work also has reported on the lack of consensus on aging biology^21^. Survey results illustrated disagreement among participants on key aspects of aging including our understanding of aging mechanisms, measurability of the aging process and treatability of aging. Our experts agreed that a multidimensional approach to endpoint selection that captures the heterogeneity of the aging processes needed. Recent efforts have also used the consensus process for the translation of biomarkers of aging in clinical applications. A panel of experts identified 6 key critical barriers to the clinical translation of biomarkers: Insufficient data sharing for biomarker development, lack of consensus on evaluation criteria for biomarkers, uncertainty on optimal timing for targeting aging, inadequate criteria for clinical implementation, challenges integrating into current healthcare model that focuses on disease treatment and lack of existing biomarkers not providing clear actionable insights for healthcare professionals^7^.The design of clinical trials allowing for surrogate biomarkers or specifically, appropriate endpoints for geroscience trials was not discussed.

It is important to acknowledge that our approach was to determine if there was consensus around a primary outcome for a geroscience trial with a prevention focus. Thus, the discussions are less relevant when considering endpoints in geroscience trials with specific more narrowly defined goals, such as using senolytics to improve physical function, or targeting aging-pathways to improve vaccine response. A strength of this study is that the experts were selected purposefully to represent views from a variety of clinical and methodologic perspectives, views that have not been well represented in other consensus building efforts. On the other hand, leaders in clinical aging research tend to be geriatricians or have a specialized focus on geriatric outcomes, and the views expressed are consistent with the emphasis on function in geriatrics. Additionally, 4 experts included had strong commercial or pharmaceutical experience; though the needs of biotechnology start-ups or pharmaceutical developers are not likely to differ significantly industry opinions may not be adequately reflected in this report.

## Future Directions / Next Steps

As the field of geroscience evolves, an aim should be to achieve greater alignment on the definition of aging, an issue essential for reaching consensus on appropriate trial endpoints. There is also a need to rationalize the components of potential composite endpoints intended for specific interventions or target populations. The geroscience hypothesis suggests that there are many aging pathways that might be targeted. It would be helpful to link perturbations in these pathways to constellations of aging phenotypes. This would facilitate the tailoring of endpoints to reflect the multifaceted nature of aging. It will also be helpful to standardize and expand the range of aging-relevant assessments collected in planned ongoing trials of interventions that are geroscience-relevant (e.g., GLP1 receptor agonists). Signals from these studies will help identify conditions and diseases linked to the therapeutic actions of the intervention. Finally, increasing dialogue between geroscience trialists and regulatory entities to find common ground on acceptable outcomes is critical to build a shared understanding of the scientific and regulatory viewpoints that if unaligned will impede progress in geroscience.

## Figures and Tables

**Figure 2:**
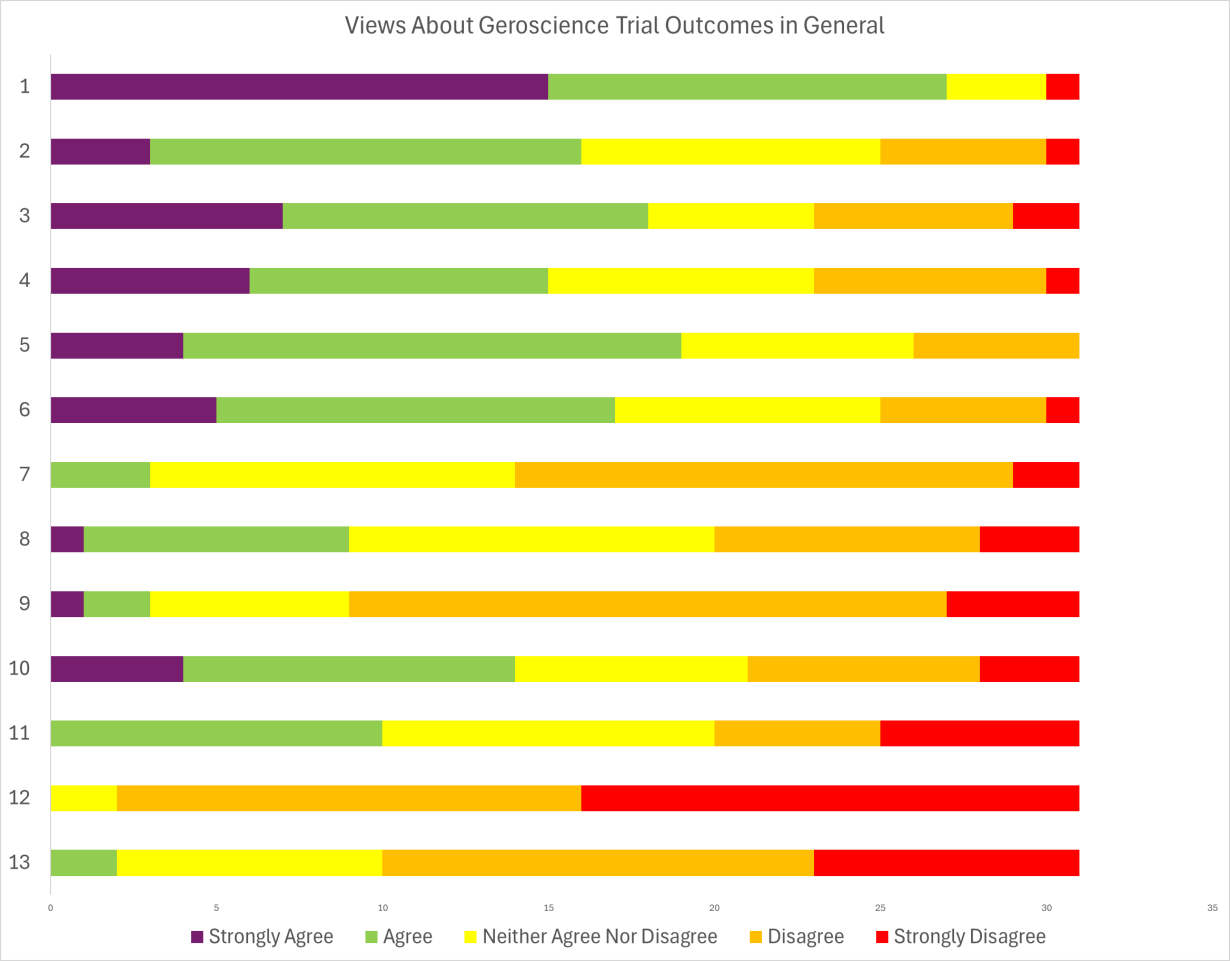
Expert views about geroscience trial outcomes in general of a clinical trial of a treatment that may slow aging results of modified Delphi process.

**Table 1:** Geroscience Trial Endpoint Expert Panel Demographics

Men / Women	21 Men, 10 Women
Clinically Trained	24, 77.4%
Currently See Patients	18 of the 24 clinically trained, 75.0%
Participated in the Design of Clinical Trials Involving Older Adults	28, 90.3%
Graduate Training in Statistics	21, 67.7%
Experience in Regulatory Process Related to Approval of Drugs	21, 67.7%
Worked in the Biology of Aging, including Model Systems of Aging	23, 74.2%
Areas Represented	Geriatric Medicine, Internal Medicine, Cardiology, Endocrinology, Clinical Neuropsychology, Clinical Pharmacology, Medical Oncology, Diabetes, Infectious Disease, Epidemiology, Gerontology, Biostatistics
Familiarity with Aging Research/Geroscience	Most of work activity is in this realm (expert) (19, 61.3%), Very familiar but not an expert (7, 22.6%), Some familiarity (5, 16.1%),

**Table 2:** Expert views on the aging process results of a modified Delphi process.

Statement	Percent Agreement (%)	Percent Neutral (%)	Percent Disagreement (%)	Mean Likert (Standard Deviation)
Aging is fundamentally a biologic phenomenon.	90.3*	0.0	9.7	1.8 (0.9)
Aging has a basis in biology which can serve as the target of an intervention.	87.1*	6.5	6.5	1.7 (0.9)
There are phenotypes that fairly represent the aging process in general.	87.1*	6.5	6.5	2.0 (0.8)
In some cases, the pathway connecting a specific biologic mechanism of aging to age-related disease is well understood.	80.6*	6.5	12.9	2.2 (0.7)
In most cases, the pathway connecting a specific biologic mechanism of aging to age-related disease is well understood.	3.2	25.8	70.1*	3.7 (0.7)
There is general agreement on what aging is.	64.5	19.4	16.1	2.4 (1.0)
Aging results from the random accumulation of biologic damage.	58.1	16.1	25.8	2.5 (1.1)
We know how to distinguish “fast” agers from “slow” agers.	35.5	48.4	16.1	2.8 (0.8)
The biologic underpinnings of aging are well understood.	29.0	25.8	45.2	2.3 (1.0)

* Consensus means greater than or equal to 66% agreed or disagreement with the statement regardless of intensity.

**Table 3.** Expert views about geroscience trial outcomes in general of a clinical trial of a treatment that may slow aging results of a modified Delphi process.

Statement	Percent Agreement (%)	Percent Neutral (%)	Percent Disagreement (%)	Mean Likert (Standard Deviation)
Outcomes used in different trials should be harmonized to the extent possible.	87.1*	9.7	3.2	1.7 (0.9)
An outcome that combines many potential age-related health conditions reflects the aging process broadly.	61.3	22.6	16.1	2.4 (0.9)
If a treatment slowed the onset of many aging-related diseases that share few risk factors other than age it would be fair to conclude that the treatment slowed aging.	58.1	16.1	25.8	2.5 (1.2)
An outcome that combines many potential age-related health conditions accounts for the heterogeneity of aging across individuals.	54.8	25.8	19.4	2.5 (1.1)
There is an index or panel of health-related measurements that is an appropriate outcome of a trial to test if a treatment slows aging.	51.6	29.0	19.4	2.6 (1.1)
An outcome that combines many age-related health conditions is hard to interpret.	48.4	25.8	25.8	2.6 (1.1)
Patient/Participant Reported Outcomes are appropriate as primary outcomes for a trial to test if a treatment slows aging.	45.2	22.6	32.3	2.8 (1.2)
There is a biomarker panel that is an appropriate primary outcome for a trial to test if a treatment slows aging.	32.3	32.3	35.5	3.2 (1.1)
An outcome developed for a trial for one intervention would be appropriate for many different interventions.	29.0	35.5	35.5	3.1 (1.0)
There is consensus regarding what patient/participant reported outcomes would be appropriate in a geroscience trial.	9.7	19.4	71.0*	3.7 (0.9)
An outcome developed for a trial of one intervention is only appropriate for that intervention.	9.7	35.5	54.8	3.5 (0.8)
There is a single health-related measure that is appropriate as the primary outcome of a trial to test if a treatment slows aging.	6.5	25.8	67.7*	3.9 (0.9)
There is a single fluid biomarker that is an appropriate primary outcome for a trial to test if a treatment slows aging.	0.0	6.5	93.5*	4.4 (0.6)

* Consensus means greater than or equal to 66% agreed or disagreement with the statement regardless of intensity.

**Table 4:** Expert views on the primary outcome(s) of a clinical trial of a treatment that may slow aging results of a modified Delphi process.

Question: The primary outcome(s) of a clinical trial of a treatment that may slow aging ...	Percent Agreement (%)	Percent Neutral (%)	Percent Disagreement (%)	Mean Likert (Standard Deviation)
would include several dimensions of health (e.g. disease, function, feelings).	83.9*	12.9	3.2	1.9 (0.7)
should reflect participant goals and desired outcomes.	74.2*	12.9	12.9	2.1 (1.0)
should be able to be assessed outside of specialized research settings.	74.2*	12.9	12.9	2.2 (0.8)
should be recognized by the FDA or other drug regulatory authority.	71.0*	22.6	6.5	2.1 (0.9)
should reflect a participant’s resilience to health stresses (e.g., infection, surgery, fracture) in some way.	71.0*	25.8	3.2	2.2 (0.7)
should include participant reported outcomes.	71.0*	16.1	12.9	2.2 (0.9)
would need to be tailored to the specific attributes of the participant population (e.g., age or functional status).	71.0*	22.6	6.5	2.1 (1.1)
should result in changes / differences noticeable to participants or their families.	67.8*	16.1	16.1	2.1 (1.1)
should be based on only objective measurements; that is things like blood pressure, or medically diagnosed disease.	35.5	12.9	51.6	3.1 (1.1)

* Consensus means greater than or equal to 66% agreed or disagreement with the statement regardless of intensity.
